# Influence of Dietary Phosphorus on the Growth, Feed Utilization, Proximate Composition, Intestinal Enzymes, and Oxidation Resistance of Sea Cucumber *Apostichopus japonicus*

**DOI:** 10.1155/2023/2266191

**Published:** 2023-04-20

**Authors:** Zhidong Song, Peiyu Li, Shunxin Hu, Caili Liu, Tiantian Hao, Xiaozhao Han

**Affiliations:** ^1^Shandong Marine Resource and Environment Research Institute, Yantai 264006, China; ^2^Yantai Key Laboratory of Quality and Safety Control and Deep Processing of Marine Food, Yantai 264006, China; ^3^Shanghai Ocean University, Shanghai 201306, China

## Abstract

Six experimental diets (crude protein 12.58%, crude fat 1.93%, and total energy 10.72 kJ/kg) containing 0.24%, 0.37%, 0.51%, 0.62%, 0.77%, and 0.89% phosphorus were formulated to evaluate dietary phosphorus requirement for sea cucumber *Apostichopus japonicus*. The feeding trial was conducted in 18 fiberglass tanks (220 L) for 63 days. Each diet was randomly assigned to triplicate tanks of 50 sea cucumbers (9.99 g) and fed once daily. With the increase of dietary phosphorus level, weight gain (WG), specific growth rate (SGR), daily feed intake (DFI), feces production ratio, the activities of amylase, alkaline phosphatase, phosphofructokinase, succinate dehydrogenase, and glutathione peroxidase as well as the contents of glutathione and glutathione oxidized significantly increased and then decreased afterwards (*P* < 0.05). *A. japonicus* fed diet with 0.63%, 0.63%, and 0.55% dietary phosphorus was estimated to yield the highest WG (11.39 g), SGR (1.09%/d), and DFI (2.55%/d) according to the quadratic regression analysis of WG, SGR, and DFI against dietary phosphorus level, respectively. The apparent digestibility of dry material and energy followed an opposite tendency. Feed efficiency, the contents of whole-body phosphorus, initially increased and then plateaued, fitting piecewise-linear models with breakpoint at 0.57% and 0.55% dietary phosphorus. Daily phosphorus intake, pyruvate kinase activity, and the ratio of glutathione and glutathione oxidized increased (*P* < 0.05) but the apparent digestibility of phosphorus, the activities of alkaline protease, aspartate transaminase, and phosphoenolpyruvate carboxykinase decreased (*P* < 0.05), responding to the increasing dietary phosphorus. Considering the present results, the optimal dietary phosphorus for *A. japonicus* is 0.57–0.63%.

## 1. Introduction

Japanese sea cucumber (*Apostichopus japonicus* (Selenka 1867)), belonging to the phylum Echinodermata, class Holothuroidea, is mainly distributed off the coasts of the North Pacific Ocean [1]. It is an economically important species as a source of seafood and ingredient in traditional medicine. Recently, the increasing market demand and prices for “bêche-de-mer” or “trepang” led to overexploitation of wild stocks worldwide and stimulated the development of commercial aquaculture of *Apostichopus japonicus* [2, 3]. Traditionally, *A. japonicus* is farmed on the ocean bottom with natural food resources. This aquaculture method has a low environmental impact, but it has low yield, high risk, and long breeding cycle. Some effective culture models, such as indoor industrial culture, pond culture, and floating raft culture, have been developed, providing greater worldwide production for the sea cucumber trade [4]. According to the recent FAO report, global aquaculture production of *A. japonicus* has more than doubled since 2005, increasing from 57,200 tonnes to 201,500 tonnes in 2020, with production growing at an annual average rate of 16.8% since then [5]. In China, *A. japonicus* aquaculture has become one of the most profitable marine culture industries, and the annual production has reached 196,500 tons in 2020, accounting for approximately 97.5% of the global production according to data from the China Fishery Statistical Yearbook [6].

The increase in *A. japonicus* aquaculture output is largely dependent upon the supply of aquaculture feed including natural seaweed and artificial feed. As a deposit feeder, sea cucumber mainly feeds on sedimentary organic matter including bacteria, protozoa, diatoms, and detritus of plants or animals in nature [7]. However, natural food is relatively insufficient in intensive aquaculture practice, and artificial feed is generally supplemented to *A. japonicus* to meet its requirement [4, 8]. Therefore, information about the quantitative nutrient requirement is required for the formulation of artificial feed. In the last twenty years, efforts towards evaluation of quantitative requirement focused on several nutrients using different nutritional assessment parameters. These studies demonstrate that *A. japonicus* has a low protein (11–17%) and lipid (1.3%) requirement ([9–11]) but high carbohydrate requirement (48.56–49.30%) [12]. In addition, several studies indicated that sea cucumber also required specific vitamins and amino acids for maximizing its growth, including ascorbic acid (100–105.3 mg/kg; [13]), *α*-tocopherol (23.1–44.0 mg/kg; [14]), riboflavin (9.73–17.9 mg/kg; [15]), lysine (0.58–0.72%; [16]), and methionine (0.76–1.19%; [17]). Up to now, there has been no reports published on phosphorus requirement of *A. japonicus*.

Phosphorus not only is a vital structural component of cell membranes and nucleic acids but also participates in many biological processes that are essential for the survival, growth, and development of aquatic animals [18–20]. In general, aquatic animals can obtain most of the minerals they require (calcium, sodium, and potassium) directly from the water (especially in sea water). Phosphorus, however, is generally found at low concentration in natural waters [21, 22]. Therefore, absorption of sufficient amounts of phosphorus from water is unlikely, making a dietary source essential for most aquatic animals [23]. A few of studies have assessed dietary phosphorus requirements for aquatic animals: 1.59–1.6% for crab *Portunus trituberculatus* [24], 1.0–1.2% for abalone *Haliotis discus* Hainan [25], 1.0% for tiger shrimp *Penaeus monodon* [26], 0.86–0.9% for Japanese seabass *Lateolabrax japonicus* [27], 0.96% for haddock *Melanogrammus aeglefinus* [28], 0.76% for yellow catfish *Pelteobagrus fulvidraco* [29], and 0.50–0.87% for Siberian sturgeon *Acipenser baerii* [30]. These results indicate dietary phosphorus requirements vary across species, and the phosphorus requirement of invertebrate is higher than vertebrate.

Some evidences show that sea cucumber can obtain available phosphorus from sediment by facilitating the transformation of organic phosphorus to inorganic phosphorus in nature [31]. However, in intensive cultivation such as land-based culture and floating cage culture, most of the phosphorus intake comes from diets including algal debris or artificial feed. Any excess of phosphorus in the diet above the minimum requirement for sea cucumber will be excreted. The excess of this element in the effluents of aquaculture systems leads to eutrophication and an adverse effect on the aquatic ecosystems. It is therefore critical to know precisely the dietary requirement of phosphorus in order to minimize excess phosphorus in feed without risking phosphorus deficiency in sea cucumber. In a fiberglass aquaculture system, we estimated the phosphorus requirement of sea cucumber by investigating the effect of dietary phosphorus on growth, diet utilization, whole body composition, digestion, and metabolism as well as oxidation resistance.

## 2. Methods and Materials

### 2.1. Preparation of Coated Sodium Dihydrogen Phosphate

Carrageenan was used to coat sodium dihydrogen phosphate to reduce phosphorus loss in water due to its low solubility loss [32]. The coated sodium dihydrogen phosphate was prepared using the methods of Li et al. [17] with some modifications: in brief, sodium dihydrogen phosphate was dissolved in distilled water and then equivalent carrageenan was added, following a complete mixing into paste. The mixture was heated in a water bath (95°C) and stirred continuously for 30 min. The coated sodium dihydrogen phosphate was freeze-dried, ground (80 mesh), and then stored at -20°C.

### 2.2. Diet Preparation

Six experimental diets were formulated to contain different levels of phosphorus (0.24%, 0.37%, 0.51%, 0.62%, 0.77%, and 0.89%) by adding coated sodium dihydrogen phosphate (Table 1). Seaweed powder (crude protein, 19.5%; crude fat, 0.82%; carbohydrate, 47.8%), fishmeal (crude protein, 67.3%; crude fat, 8.4%), shrimp powder (crude protein, 58%; crude fat, 14%), and yeast powder (crude protein, 53%; crude fat, 3.6%) were ground and sieved (80 mesh). All dry ingredients were weighed in the proportions as presented in Table 1 and then mixed thoroughly (20 min) in a Patterson-Kelley twin shell® Batch V-mixer (Patterson-Kelley Co., Inc., East Stroudsburg, PA). The coated sodium dihydrogen phosphate and vitamin premix were added by the progressive enlargement method. Subsequently, fish oil and distilled water were appended to the dry ingredients until homogenous in mixer and then cold-extruded and sliced into strips (1 cm × 0.5 cm × 0.08 cm) (MZLP400, Anyang Jimke Energy Machinery Co., Ltd.). These strip feeds were air-dried until moisture was lower than 6%, then kept in vacuum-packed bags and stored at -20°C.

### 2.3. Feeding Trial

The feeding trial was conducted in a hatchery (Dongying, China). *A. japonicus* was obtained from Shandong Anyuan Aquaculture Co. Ltd. (Penglai, China). After acclimation to the experimental conditions for 2 weeks, 900 sea cucumbers with an average initial weight of 9.99 ± 0.02 g were randomly assigned into 18 fiberglass tanks (L-100 cm; W-50 cm; H-80 cm). Each diet was randomly fed to sea cucumbers in triplicate tanks for 10 weeks. The feeding amount was 10% of wet weight daily (recalculated and adjusted every two weeks) in order to collect enough feces for analyses of apparent digestibility and fecal production rate as soon as possible. Feces and uneaten feed residues were collected by siphoning before the next feeding and dried at 60°C to a constant weight for further analysis [4]. Some intact feces were picked out for further digestibility analysis. The weight of uneaten feed was assessed by the leaching ration of diets in water [33]. During the trail, the water temperature was maintained at 18.0 ± 0.5°C by seawater source heat pump system (Zhuoren Air Conditioning Equipment Co., Ltd, Shandong, China). The pH was maintained at 7.1 ± 0.1 by adjusting the water exchange rate (300%/d), and dissolved oxygen was kept higher than 6.0 mg/L by oxygen pump (2HB520-7HH57, Weisida Electromechanical Co., Ltd, Huzhou, China). These water quality parameters were monitored daily during the trial.

### 2.4. Sample Collection and Growth Calculation

At the end of the feeding trial, sea cucumbers in each tank were bulk-weighed. Then, 13 sea cucumbers from each tank were weighed individually and dissected. Intestines were weighed and quickly frozen in liquid nitrogen. Pooled intestines of each replicate tank were homogenated with physiological saline solution (0.7% NaCl) and then centrifuged at 8,000 rpm for 10 min. The supernatant was divided into aliquots of 400 *μ*l in 1.5 ml centrifugal tube and stored at -80°C until the enzyme activity analysis.

The growth parameters and diet utilization were calculated according to the following formulas:

(1) $$\begin{array}{c} \text{Weight gain }\left(\text{WG},\text{g}\right)=\text{final weight }\left(\text{g}\right)–\text{initial weight }\left(\text{g}\right); \end{array}$$

(2) $$\begin{array}{c} \text{Specific growth rate }\left(\text{SGR},\%/\text{d}\right)=\frac{\left(\text{Ln final weight}–\text{Ln initial weight}\right)}{\text{days of experiment}}\times 100; \end{array}$$

(3) $$\begin{array}{c} \text{Daily feed intake }\left(\text{DFI},\%/\text{d}\right)=\frac{\text{dry weight of consumed feed}\left(\text{g}\right)}{\left[\left(\text{initial weight}+\text{final weight}\right)/2\times t\right]}\times 100; \end{array}$$

(4) $$\begin{array}{c} \text{Daily phosphorus intake }\left(\text{DPI},\%/\text{d}\right)=\text{Daily feed intake}\times \text{phosphorus content}; \end{array}$$

(5) $$\begin{array}{c} \text{Feces production rate }\left(\text{FPR},\%/\text{d}\right)=\frac{\text{dry feces weight}}{\left[\text{days}\times \left(\text{initial weight}+\text{final weight}\right)/2\right]}\times 100; \end{array}$$

(6) $$\begin{array}{c} \text{Feed efficiency }\left(\text{FE}\right)=\frac{\text{weight gain}}{\text{weight of consumed feed}}; \end{array}$$

(7) $$\begin{array}{c} \text{Survival rate}\left(\text{SR},\%\right)=\frac{\text{final sea cucumber number}}{\text{initial sea cucumber number }}\times 100; \end{array}$$

(8) $$\begin{array}{c} \text{Apparent digestibility coefficient of dry material }\left({\text{AD}}_{\text{m}},\%\right)=\left(\frac{1‐\text{dietary acid}‐\text{insoluble ash content}}{\text{fecal acid}‐\text{insoluble ash content}}\right)\times 100; \end{array}$$

(9) $$\begin{array}{c} \text{Apparent digestibility coefficients of protein }\left({\text{AD}}_{\text{p}},\%\right),\text{fat }\left({\text{AD}}_{\text{f}},\%\right),\text{energy }\left({\text{AD}}_{\text{e}},\%\right),\text{and phosphorus }\left({\text{AD}}_{\text{pi}},\%\right)=100-100\times \frac{\left(\text{fecal nutrient content}\times \text{dietary acid}-\text{insoluble ash content}\right)}{\left(\text{dietary nutrient content}\times \text{fecal acid}-\text{insoluble ash content}\right)}; \end{array}$$

### 2.5. Proximate Composition Analysis

Proximate compositions of diets, body walls, and feces were analyzed according to the standard methods of AOAC [34]. Moisture was determined by drying samples at 105°C for 2 h in oven (Binder FD-S56, BINDER GmbH, German). Crude protein (*N* × 6.25) was determined using Kjeldahl nitrogen analyzer (Kjeltec 8100, FOSS Analytical Co., Ltd., Denmark) following an acid digestion (DT 220 Digestor, FOSS Analytical Co., Ltd., China). Crude fat was analyzed by the ether extraction method using fat analyzer (SOX-406, Jinan Hanon Instruments Co., Ltd., China). Crude ash was determined using a muffle furnace (Linder/blue M1100, Thermo Fisher Scientific Co., Ltd., China) at 550°C for 6 h. Total energy was measured with an automatic calorimeter (IKA C6000, Aika Instrument and Equipment Co., Ltd., Guangzhou). Acid-insoluble ash was determined according to the method of Khalil, et al. [35]. Total phospholipids were separated from total lipid with a silica gel-based solid-phase extraction column, and then quantitation was performed by hydrophilic interaction HPLC coupled to evaporative light-scattering detection using a quaternary separation method [36]. The samples of whole body and diets were digested with HNO_3_ in microwave oven (ZUOT-SYS-WBL Shanghai Satian Precision Co., Ltd, China), and total phosphorus contents were analyzed using the vanadomolybdophosphoric acid method with a spectrophotometer (U-3900H, Hitachi Co., Ltd., Japan) set at a wavelength of 420 nm [37].

### 2.6. Activities Analyses of Intestinal Enzymes

The activities of acid and alkaline proteases were assayed using the Folin-Ciocalteu's reagent according to methods of Cui et al. [38]. Lipase activity was determined according the method of Massadeh and Sabra [39] using *p*-nitrophenyl palmitate (*p*NPP) (Sigma, USA) as substrate. Amylase activity was analyzed by measure absorbance value change of starch-iodine reaction solution at the wavelength of 660 nm according the method of Al-Qodah et al. [40]. The ALP and ACP activities are estimated by mixing crude enzyme extract with a reaction of disodium phenyl phosphate buffer at pH 10.5 and 4.9 and followed by estimation of absorbance of the resultant chromogenic solution at 520 nm, respectively [41].

Superoxide dismutase (SOD) was measured with a microplate reader according to the method of Peskin and Winterbourn [42]. One unit (U/mg prot) of SOD activity is defined as the amount of enzyme in 1 mg tissue protein that inhibits the rate of reduction of cytochrome C by 50% in a coupled system, using xanthine and xanthine oxidase at pH 7.8 at 37°C. Catalase (CAT) activity was determined using a spectrophotometric assay of hydrogen peroxide based on formation of its stable complex with ammonium molybdate as described in detail by Goth [43]. Glutathione peroxidase (GSH-Px) activity was measured by DTNB (5,5′-dithiobis-(2-nitrobenzoic acid)) method as described by Fukuzaw and Tokumur [44]. One unit (U/mg prot) of GSH-Px activity is defined as the amount of enzyme that catalyzes the oxidation by H_2_O_2_ of 1.0 *μ*mol of reduced glutathione to oxidized glutathione per minute at pH 7.0 at 37°C. Malondialdehyde (MDA) was determined using the acid extraction TBA (thiobarbituric acid) method as reported by Lynch and Frei [45], and results were expressed as nmol MDA per mg of tissue protein (nmol/mg prot). GSH was determined spectrophotometrically according to Drukarch et al. [46], and the content was expressed as *μ*mol GSH per gram of tissue protein (*μ*mol/g prot). Protein content was determined by the method of Lowry et al. [47].

### 2.7. Statistical Analysis

The Software SPSS 12.0 microcomputer software package (SPSS, Chicago, IL, USA) was used for all statistical evaluations. A homogeneity test for variance was conducted. All data were subjected to one-way analysis of variance (ANOVA) followed by Tukey's test. Differences were regarded as significant when *P* < 0.05. Data are expressed as mean and standard deviation with pooled SE. Nonlinear regression analysis was used to describe the relationship between growth, diet utilization, and whole-body phosphorus content.

## 3. Results

### 3.1. Growth Performance

**Figure 1:**
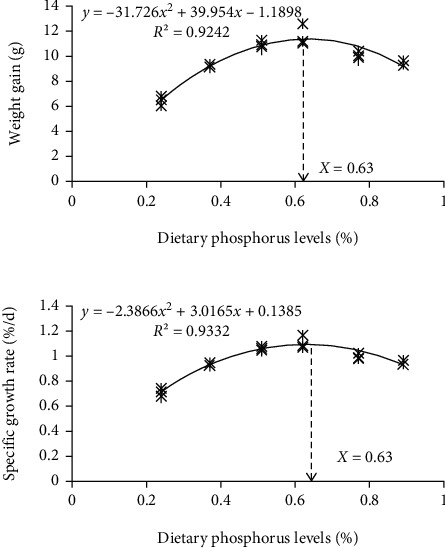
Quadratic regression analyses of weight gain and specific growth rate (*y*-axis) in sea cucumber fed diets with graded phosphorus levels (*x*-axis) (*n* = 3). The predicted dietary phosphorus level is 0.63% for maximum growth.

As summarized in Table 2, there was no difference in survival rate (SR) among dietary treatments (*P* > 0.05). Final weight (FW), weight gain (WG), and specific growth rate (SGR) significantly increased with dietary phosphorus level increasing from 0.24% to 0.62%, but decreased thereafter (*P* < 0.05). The higher FW, WG, and SGR were observed in *A. japonicus* fed 0.51% and 062% dietary phosphorus than in those fed other diets (*P* < 0.05). *A. japonicus* receiving diet containing 0.24% phosphorus showed the lowest WG and SGR (*P* < 0.05). Application of quadratic regression analyses to the WG and SGR provided an estimate of 0.63% dietary phosphorus for optimum growth, with the predicted the maximum WG (11.39 g) and SGR (1.09%/d) (Figure 1).

### 3.2. Diet Utilization

**Figure 2:**
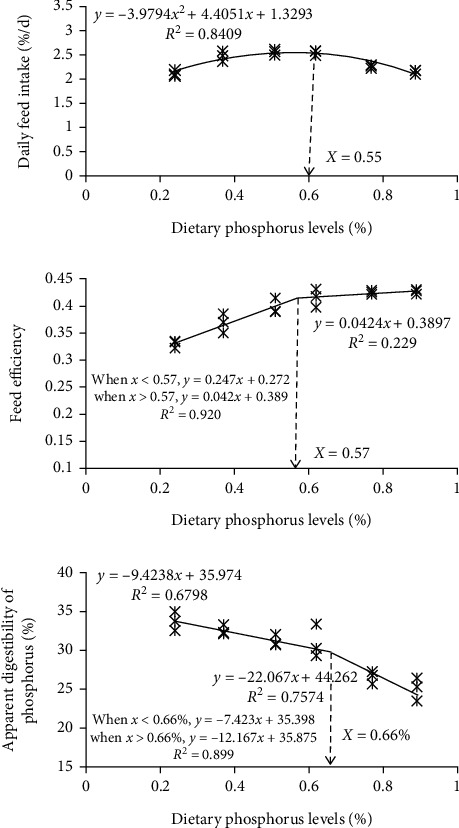
The quadratic regression analysis of daily feed intake and piecewise regression analyses of feed efficiency and apparent digestibility of phosphorus (*y*-axis) in sea cucumber fed diets with graded phosphorus levels (*x*-axis) (*n* = 3), respectively. The predicted dietary phosphorus levels are 0.55% for the maximum feed intake, 0.57% for the optimal feed efficiency, and 0.66% for optimal phosphorus digestibility.

Diet utilization was presented in Table 3. AD_p_ and AD_f_ were not affected by dietary phosphorus levels (*P* > 0.05). DFI and FPR increased to the peak values (2.55%/d and 2.08%/d, respectively) as dietary phosphorus increased from 0.24% to 0.51%, and then both decreased (*P* < 0.05). However, AD_m_ and AD_e_ followed an opposite tendency with the lowest AD_m_ (17.56%) and AD_e_ (25.85%) observed in *A. japonicus* fed 0.51% phosphorus. FE significantly increased to 0.41 with dietary phosphorus increasing up to 0.62% and then reached a plateau within 0.41–0.43 (*P* < 0.05). DPI gradually increased while AD_pi_ decreased with the increase of dietary phosphorus (*P* < 0.05). The quadratic regression analysis was applied to illustrate the DFI response, predicting dietary phosphorus of 0.63% for maximum DFI. The piecewise regression analyses were applied to FE and AD_pi_, locating the breakpoint (0.57% and 0.66%) of dietary phosphorus levels (Figure 2).

### 3.3. Activities Analyses of Intestinal Enzymes

As summarized in Table 4, the activities of ACPT, ALT, HK, and CS were not affected by dietary phosphorus (*P* > 0.05). The activities of AKPT, AST, and PEPCK decreased significantly but PK activity increased (*P* < 0.05) with dietary phosphorus increasing. The activities of ALP, PFK, and SDH first increased and then decreased, responding to the increasing dietary phosphorus. The highest activities of ALP and SDH were observed at dietary phosphorus of 0.62% and the highest PFK activity at dietary phosphorus of 0.51%. *A. japonicus* fed 0.51–0.62% phosphorus had similar ALP activities (*P* > 0.05), and *A. japonicus* fed 0.51–0.77% phosphorus had similar SDH activities (*P* > 0.05), which were significantly higher than those fed other diets (*P* < 0.05). The amylase acitivity firstly increased to 19.60 U/mg prot with dietary phosphorus increasing up to 0.62%, but decreased slightly thereafter.

### 3.4. Proximate Composition of Whole Body

**Figure 3:**
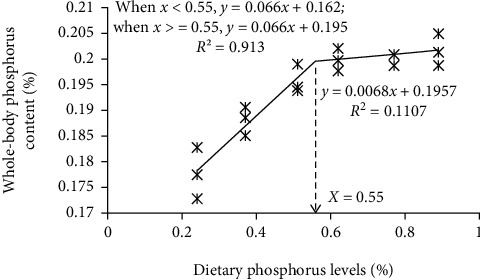
A piecewise regression analysis of whole-body phosphorus content (*y*-axis) in sea cucumber fed diets with graded phosphorus levels (*x*-axis) (*n* = 3). The predicted dietary phosphorus level is 0.55% for the optimal whole-body phosphorus deposition.

As presented in Table 5, there were no significant differences in the contents of crude protein, crude fat, and crude ash of whole body among all groups (*P* > 0.05). The contents of phospholipid and whole-body phosphorus increased with the dietary phosphorus increasing up to 0.51% (*P* < 0.05) and fluctuated within 0.56–0.59% and 1.96–2.02 mg/g, respectively, as dietary phosphorus further increased (*P* > 0.05). A piecewise regression analysis was used to describe the relationship between whole-body phosphorus content and dietary phosphorus level, with the predicted optimal phosphorus level of 0.55% for the whole-body phosphorus deposition (Figure 3).

### 3.5. Intestinal Oxidation Resistance

As presented in Table 6, the intestinal of CAT activity and MDA content was not affected by dietary phosphorus (*P* > 0.05). The activities of GSH-Px and SOD showed a quadratic response to the incremental increase of dietary phosphorus (*P* < 0.05), with the highest activities of GSH-Px (33.52 U/mg prot) and SOD (5.26 U/mg prot) occurring at dietary phosphorus levels of 0.62% and 0.51%, respectively. The contents of GSH and GSSG exhibited an ascending trend with dietary phosphorus increasing from 0.24% to 0.51% and then followed by a significant reduction (*P* < 0.05). The contents of GSH and GSSG were highest in *A. japonicus* fed 0.51% phosphorus and lowest in those fed 0.24% phosphorus (*P* < 0.05). The ratio of GSH/GSSG was significantly elevated in *A. japonicus* fed diets containing 0.77% and 0.89% phosphorus as compared to those fed other diets (*P* < 0.05).

## 4. Discussion

### 4.1. Effects of Dietary Phosphorus on Growth of *A. japonicus*

During the feeding trial, *A. japonicus* grew from 9.99 g to 14.67–21.60 g with an acceptable SGR (0.71–1.11%/d), as compared to the growth data from other studies [17, 48–50]. These positive growth responses fit quadratic regression models and thus confirm that phosphorus deficiency or excess could hinder the growth of *A. japonicus* as reported in a previous study on tiger shrimp [26]. Therefore, an appropriate supply of dietary phosphorus is necessary for *A. japonicus* in aquaculture. On the other hand, the estimated dietary phosphorus requirement from these quadratic regression analyses appears lower than the published reports on fish (0.72–1.57%, [28, 51–53]), shrimp (1–2%, [26, 54–57]), crab (1.59–1.68%, [24]), and abalone (1–1.2%, [25]), implicating that sea cucumber may have an specific phosphorus acquisition mechanism. This is proved by a recent physiological findings indicated the phosphatase exhibited comparable activity levels in the respiratory tree segments of sea cucumber *Isostichopus badionotus* [58]. Another evidence from an early P^32^ isotope study also revealed that sea cucumber absorbed a limited amount of phosphorus from the environment through the integumentary surface and respiratory tree [59]. The phosphorus obtained by this additional way cannot completely meet the growth needs of *A. japonicus*, but reduce its dietary requirement.

### 4.2. Effects of Dietary Phosphorus on Diet Utilization of *A. japonicus*

Feeding response to dietary phosphorus varies across species, and phosphorus deficiency symptom generally manifests as appetite loss and low feed efficiency (reviewed by [18, 28, 60]). Therefore, in the present study, feeding diet containing 0.24% phosphorus resulted in low daily feed intake and feed efficiency. However, it was noted that with dietary phosphorus increasing, DFI and FPR showed a quadratic variation while FE showed a piecewise linear variation, fitting different regression models. This indicated that dietary phosphorus exerted different effects on the daily feeding intake, daily phosphorus intake, and feed efficiency. Aquatic animal can control the nutritional equilibrium by altering their feeding response including feeding intake and feed efficiency, as reported on gibel carp *Carassius auratus Gibelio* [61] and sea cucumber *Australostichopus mollis* [62]. In the present study, the feeding response result indicated that *A. japonicus* could sense body phosphorus status and control phosphorus intake by regulating feed intake. According to quadratic regression analysis of DFI, dietary phosphorus of 0.55% was optimal for maximum feed intake. In addition, the change point of slope estimated by a piecewise linear regression analysis of FE represented a change of body phosphorus status, suggesting that *A. japonicus* feeding >0.57% dietary phosphorus could allocate more phosphorus for supporting growth than that fed low phosphorus.

Apparent digestibility is not only as an estimate of digestive efficiency. Generally, there is a trade-off between intake and digestive efficiency. Higher diet intake equates to faster digesta transit, which can result in a lower digestive efficiency [63]. Therefore, the reduction of AD_m_ and AD_e_ of *A. japonicus* in the present study may result from high feed intake, which agrees with the report on turtle *Pelodiscus sinensis* [64]. In addition, apparent digestibility was also indicator of the balance of nutrient deposition and excretion. In the present study, AD_pi_ decreased proportionately with dietary phosphorus increasing, which was consistent with the findings on largemouth bass *Micropterus salmoides* [52]. However, a change point (0.66% dietary phosphorus) was detected by a piecewise linear regression model in the relationship via a change in slope. This change point represents the threshold of dietary phosphorus, above which body phosphorus status of *A. japonicus* is altered and the proportion of fecal phosphorus becomes higher. This agrees with the studies on rainbow trout [65] and tiger shrimp [26] and suggests that phosphorus excretion occurs when the phosphorus intake is above the requirement level. Therefore, dietary phosphorus should be controlled within the range of 0.57–0.66%.

### 4.3. Effects of Dietary Phosphorus on Intestinal Enzymes of *A. japonicus*

The whole digestion process primarily relies on the types and activities of digestive enzymes [66, 67]. Several studies reported that *A. japonicus* could modulate their digestive enzyme activities in response to different diet qualities, suggesting its digestive flexibility [49, 68, 69]. In the present study, dietary phosphorus stimulated the activities of lipase and amylase, which agreed with the results reported on coho salmon *Oncorhynchus kisutch* [70] and red swamp crayfish *Procambarus clarkia* [71]. However, intestinal protease exhibited a descending trend as dietary phosphorus increased, suggesting that dietary phosphorus reduced protein digestion. Induction and secretion of phosphatase is one of the important adaptive responses of the animal to low phosphorus status. Studies with abalone [25] and turtle *Pelodiscus sinensis* [64] demonstrated that the tissue activity of alkaline phosphatase is negatively correlated with dietary phosphorus levels. However, the present study showed that dietary phosphorus-stimulated intestinal phosphatase activity as dietary phosphorus level increased up to 0.62%. This stimulatory effect may be associated with the increased feeding intake because feed intake is considered as a major driver of alkaline phosphatase activity (reviewed by [72]).

In line with the abovementioned protease, the activities of two intestinal transaminases of *A. japonicus* were decreased by dietary phosphorus in the present study. Similar finding was also reported on catfish *Silurus asotus* [73], suggesting that dietary phosphorus reduced oxidation of amino acids. On the other hand, dietary phosphorus significantly stimulated the activities of PK and PFK but reduced PEPCK activity, which agreed with the recent findings on blunt snout bream *Megalobrama amblycephala* [74] and swimming crab *Portunus trituberculatus* [24], indicating an enhanced glycolysis coupling with a depressed gluconeogenesis. In addition, the membrane-bound succinate dehydrogenase (SDH) contributes to the establishment of the mitochondrial membrane potential and ATP synthesis [75]. This elevated SDH activity in *A. japonicus* fed 0.37–0.62% phosphorus suggested that appropriate amount of dietary phosphorus facilitated the tricarboxylic acid cycle and energy production. Taken collectively, dietary phosphorus altered *A. japonicus* intestinal metabolism and allowing more energy produced for growth, which may explain the growth result.

### 4.4. Effects of Dietary Phosphorus on Proximate Composition of *A. japonicus*

The whole-body phosphorus content has been commonly used as an indicator of dietary phosphorus status. Signs of phosphorus deficiency were generally characterized by low whole-body phosphorus [51, 76]. Our results showed the content of whole-body phosphorus significantly increased in response to the increasing dietary phosphorus, which was consistent with the research results on yellow catfish *Pelteobagrus fulvidraco* [51], pejerrey fingerlings *Odontesthes bonariensis* [77], stinging catfish *Heteropneustes fossilis* [78], largemouth bass [52], and swimming crab [24]. The piecewise regression analysis located the breakpoint of whole-body phosphorus at a dietary phosphorus of 0.57%, lower than the growth requirement of 0.63%. This may be attributed to a fact that a dynamic “phosphorus pool” exists in organisms, playing an important role in controlling phosphorus homeostasis [79]. Sea cucumber preferentially fill the “phosphorus pool” with exogenous phosphorus and then allocate for growth.

Phospholipid is an essential component of the cell membrane and predominate in the composition of lipids in sea cucumber [80]. The increase in phospholipids representing an important adaptive strategy for marine invertebrates in resilience to environmental stress [81–83]. In the present study, dietary phosphorus prompted the biosynthesis of phospholipids and thus explain the increased phospholipids content. This contradicted the findings of another study on the growth of Japanese flounder [84] and implicated that sea cucumber may develop phospholipid synthesis ability. More researches are needed to verify this speculation.

### 4.5. Effects of Dietary Phosphorus on Oxidation Resistance of *A. japonicus*

Reactive oxygen species are byproducts of normal mitochondrial metabolism and homeostasis [85]. However, excessive ROS can induce cell damage (oxidative stress) [86]. Sea cucumber, similarly to other invertebrates, is endowed with efficient ROS-scavenging mechanisms [87, 88]. Superoxide dismutase (SOD) and glutathione peroxidase (GSH-px) are two major antioxidant enzymes, involved in response of *A. japonicus* to environmental stress and nutritional stimulation [31, 89, 90]. Dietary phosphorus deficiency downregulated the mRNA levels and activities of antioxidant enzymes in fish [91]. In the present study, the activities of SOD and GSH-Px as well as GSSH concentration were significantly elevated by the moderate level (0.37–0.77%) of dietary phosphorus, which agreed with the findings in juvenile Jian carp [92], grass carp [93], and juvenile snakehead [94]. This suggests that appropriate amount of dietary phosphorus promote synthesis of SOD and GSH-Px, protecting the intestine from free radicals and keeping MDA at a low level. Therefore, dietary phosphorus levels should control within the range of 0.37–0.77% without negative impacts on *A. japonicus*.

## 5. Conclusion

In summary, it is necessary to provide appropriate dietary phosphorus in *A. japonica* aquaculture, though *A. japonica* has a relatively low requirement. Dietary phosphorus deficiency hindered the growth of sea cucumber, but excessive phosphorus increased fecal phosphorus excretion, which exerted a potential negative impact on the aquaculture environment. Considering the results in the current study, the optimal dietary phosphorus for sea cucumber were 0.57–0.63%. This provides an important reference for feed formulators to develop nutritionally balanced commercial diets that promote optimal growth and health of sea cucumber with the minimal impact on the environment. Also, the present result in terms of diet utilization provides a reference for calculating the maximum tolerable daily or weekly feed consumption/intake, to reduce feed waste. In the future, a strategy for improving phosphorus utilization in commercial feeds for sea cucumber is necessary, not only for economic but also for environmental reasons.

## Figures and Tables

**Table 1:** Formulation and proximate composition of the experimental diets.

Ingredients (% dry basis)	Dietary phosphorus level (%)					
	0.24	0.37	0.51	0.62	0.77	0.89
Seaweed powder^1^	23.5	23.5	23.5	23.5	23.5	23.5
Fishmeal^2^	8	8	8	8	8	8
Shrimp powder^3^	3	3	3	3	3	3
Yeast powder^4^	0.5	0.5	0.5	0.5	0.5	0.5
Fish oil^5^	1	1	1	1	1	1
Carrageenan^6^	5	5	5	5	5	5
Microcrystalline cellulose^7^	8	7	6	5	4	3
Sodium dihydrogen phosphate^8^	0	1	2	3	4	5
Sea mud^9^	50	50	50	50	50	50
Vitamin premix^10^	1	1	1	1	1	1
Total	100	100	100	100	100	100

Proximate composition (measured value, %)						
Crude protein	12.44	12.45	12.63	12.78	12.52	12.66
Crude fat	1.94	1.92	1.98	1.90	1.91	1.92
Ash	56.17	56.03	56.24	56.17	56.26	56.14
Total phosphorus	0.24	0.37	0.51	0.62	0.77	0.89
Energy (kJ/kg)	10.65	10.70	10.72	10.74	10.75	10.78

^1^A mixture of *Spiraea thunbergii*, *L. japonica*, *S. polycystum*, and *Ulva lactuca* was obtained from Rongcheng Haihao Biotechnology Co., Ltd. Crude protein, 19.5%; crude fat, 0.82%; carbohydrate, 47.8%. ^2^Tongri Food Co., Ltd., Tsingdao, China. Crude protein, 67.3%; crude fat, 8.4%. ^3^Xingyi Marine Biological Engineering Co., Ltd., Guangdong, China. Crude protein, 58%; crude fat, 14%. ^4^Angel yeast limited by Share Ltd., Hubei, China. Crude protein, 53%; crude fat, 3.6%. ^5^Yaheng Biological Technology Co. Ltd., Shandong, China. Purity, 99%. ^6^Linghu Xinwang Chemical Co., Ltd., Zhejiang, China. Carbohydrate, 97.8%. ^7^Weifang Haizhiyuan Chemical Co., Ltd., Shandong, China. Purity, 94%. ^8^Shijiazhuang Spring Letter Biological Technology Co., Ltd., Hebei, China. Purity, 99%. ^9^Collected from cage culture area in Changdao sea area (Penglai, China), phosphorus content, 0.018%. ^10^Vitamin premix(mg/kg): retinol acetate, 38.0; cholecalciferol, 13.2; a-tocopherol, 210.0; thiamin, 115.0; riboflavin, 380.0; pyridoxine, 88.0; pantothenic acid, 368.0; niacin acid, 1030.0; biotin, 10.0; folic acid, 20.0; vitamin B_12_, 1.3; inositol, 4000.0; ascorbic acid, 500.0.

**Table 2:** Growth performance of *A. japonicus* fed experimental diets with different phosphorus levels.

	Dietary phosphorus level (%)						Pooled SE
	0.24	0.37	0.51	0.62	0.77	0.89	
IW^1^ (g)	9.99 ± 0.02	9.99 ± 0.05	10.01 ± 0.05	9.94 ± 0.19	10.00 ± 0.00	10.01 ± 0.04	0.018
FW^2^ (g)	16.47 ± 0.38^a^	19.31 ± 0.13^b^	21.02 ± 0.25^c^	21.60 ± 0.93^c^	20.14 ± 0.30^b^	19.56 ± 0.19^b^	0.408
WG^3^ (g)	6.47 ± 0.37^a^	9.32 ± 0.12^b^	11.01 ± 0.27^d^	11.65 ± 0.85^d^	10.14 ± 0.30^c^	9.55 ± 0.20^bc^	0.408
SGR^4^ (%/d)	0.71 ± 0.03^a^	0.94 ± 0.01^b^	1.06 ± 0.02^d^	1.11 ± 0.05^d^	1.00 ± 0.02^c^	0.96 ± 0.02^bc^	0.031
SR^5^ (%)	87.78 ± 3.85	92.22 ± 6.94	91.11 ± 6.94	90.00 ± 3.33	91.11 ± 3.33	90.00 ± 5.77	1.094

Notes: values in the same row with different superscript letters show significant difference (*P* < 0.05). ^1^Initial weight (IW, g); ^2^final weight (FW, g); ^3^weight gain (WG, g); ^4^specific growth rate (SGR, %/d); ^5^survival rate (SR, %).

**Table 3:** Diet utilization of *A. japonicus* fed diets with different phosphorus levels.

	Dietary phosphorus level (%)						Pooled SE
	0.24	0.37	0.51	0.62	0.77	0.89	
DFI^1^ (%/d)	2.12 ± 0.05^a^	2.47 ± 0.10^c^	2.55 ± 0.06^c^	2.54 ± 0.05^c^	2.26 ± 0.04^b^	2.16 ± 0.04^ab^	0.051
FPR^2^ (%/d)	1.65 ± 0.17^ab^	2.01 ± 0.06^c^	2.08 ± 0.06^c^	2.04 ± 0.11^c^	1.77 ± 0.08^b^	1.57 ± 0.08^a^	0.052
FE^3^	0.33 ± 0.01^a^	0.37 ± 0.02^a^	0.40 ± 0.01^b^	0.41 ± 0.02^bc^	0.43 ± 0.00^c^	0.43 ± 0.00^c^	0.009
DPI^4^ (10^−2^%/d)	0.51 ± 0.01^a^	0.91 ± 0.04^b^	1.30 ± 0.03^c^	1.58 ± 0.03^d^	1.74 ± 0.03^e^	1.92 ± 0.04^f^	0.118
AD_m_^5^ (%)	33.70 ± 1.18^c^	32.52 ± 0.62^bc^	31.15 ± 0.72^b^	30.92 ± 2.14^b^	26.51 ± 0.78^a^	25.05 ± 1.44^a^	0.800
AD_p_^6^ (%)	18.74 ± 1.58^ab^	18.26 ± 1.25^ab^	17.56 ± 1.52^a^	19.16 ± 0.91^ab^	19.98 ± 1.48^ab^	20.73 ± 0.68^b^	0.359
AD_f_^7^ (%)	22.76 ± 1.36	22.06 ± 1.27	23.35 ± 0.77	23.78 ± 1.99	22.51 ± 2.08	22.83 ± 1.79	0.347
AD_pi_^8^ (%)	34.24 ± 3.70	32.92 ± 1.52	31.78 ± 1.42	33.50 ± 1.18	34.09 ± 2.45	34.63 ± 1.32	0.480
AD_e_^9^ (%)	29.47 ± 0.83^bc^	28.51 ± 0.98^ab^	25.85 ± 0.71^a^	27.99 ± 0.87^ab^	30.90 ± 3.19^bc^	32.35 ± 2.23^c^	0.557

Notes: values in the same row with different superscript letters show significant difference (*P* < 0.05). ^1^Daily feed intake (DFI, %/d); ^2^feces production rate (FPR, %/d); ^3^feed efficiency (FE); ^4^daily phosphorus intake (DPI, %/d); ^5^apparent digestibility coefficients of dry material (AD_m_,%); ^6^apparent digestibility coefficients of protein (AD_p_,%); ^7^apparent digestibility coefficients of fat (AD_f_,%); ^8^apparent digestibility coefficients of phosphorus (AD_pi_%); ^9^apparent digestibility coefficients of energy (AD_e_%).

**Table 4:** Intestinal enzymes of sea cucumber fed diets with different phosphorus levels.

	Dietary phosphorus level (%)						Pooled SE
	0.24	0.37	0.51	0.62	0.77	0.89	
Digestive enzymes							
ACPT^1^ (U/mg prot)	0.56 ± 0.03	0.55 ± 0.06	0.59 ± 0.10	0.49 ± 0.02	0.51 ± 0.02	0.55 ± 0.03	0.013
AKPT^2^ (U/mg prot)	243.60 ± 21.78^c^	220.63 ± 6.20^b^	199.25 ± 4.86^b^	156.35 ± 9.73^a^	150.88 ± 12.25^a^	138.83 ± 12.26^a^	4.317
Amylase (U/mg prot)	15.97 ± 0.75^a^	17.92 ± 0.64^ab^	19.60 ± 1.29^bc^	21.32 ± 2.63^c^	20.84 ± 1.82^bc^	19.71 ± 1.79^bc^	0.546
Lipase (U/g prot)	4.97 ± 0.83^a^	7.61 ± 0.47^b^	6.95 ± 0.16^b^	7.35 ± 0.57^b^	7.44 ± 0.93^b^	6.97 ± 1.47^b^	0.273

Metabolic enzymes (U/g prot)							
ALP^3^	52.20 ± 1.80^a^	68.08 ± 1.73^b^	83.41 ± 1.02^cd^	84.30 ± 2.64^d^	80.60 ± 2.02^c^	66.68 ± 1.56^b^	2.807
AST^4^ (×10^1^)	72.82 ± 5.51^d^	63.73 ± 2.50^cd^	53.66 ± 4.69^b^	56.26 ± 6.74^bc^	46.87 ± 4.20^ab^	39.94 ± 6.73^a^	2.800
ALT^5^ (×10^2^)	38.58 ± 2.44	37.65 ± 3.88	39.49 ± 2.57	40.81 ± 2.18	38.57 ± 3.78	37.83 ± 0.96	61.512
HK^6^	67.72 ± 3.42	67.40 ± 4.86	65.70 ± 4.73	63.45 ± 5.02	64.21 ± 1.90	62.91 ± 3.93	0.878
PK^7^	32.62 ± 5.12^a^	33.52 ± 2.28^a^	33.83 ± 4.04^a^	42.46 ± 1.83^b^	43.76 ± 2.77^b^	45.72 ± 5.40^b^	1.518
PFK^8^	8.09 ± 2.07^a^	9.21 ± 0.86^ab^	12.01 ± 1.18^b^	11.96 ± 2.80^b^	10.90 ± 1.18^ab^	10.57 ± 0.68^ab^	0.417
PEPCK^9^	42.16 ± 6.59^c^	40.83 ± 7.24^c^	36.12 ± 6.13^bc^	26.39 ± 4.89^ab^	26.16 ± 5.22^ab^	24.35 ± 1.56^a^	2.087
CS^10^	5.32 ± 0.44	5.51 ± 1.31	4.88 ± 0.65	4.84 ± 0.53	5.14 ± 0.41	4.96 ± 0.33	0.149
SDH^11^	5.16 ± 0.81^a^	5.31 ± 0.34^a^	5.90 ± 0.34^b^	6.35 ± 0.35^b^	6.25 ± 0.63^b^	5.23 ± 0.20^a^	0.153

Notes: values in the same row with different superscript letters show significant difference (*P* < 0.05). ^1^Acid protease (ACPT, U/mg prot); ^2^alkaline protease (AKPT, U/mg prot); ^3^alkaline phosphatase (ALP, U/g prot); ^4^aspartate transaminase (AST, U/g prot); ^5^alanine transaminase (ALT, U/g prot); ^6^hexokinase (HK, U/g prot); ^7^pyruvate kinase (PK, U/g prot); ^8^phosphofructokinase (PFK,U/g prot); ^9^phosphoenolpyruvatecarboxykinase (PEPCK, U/g prot); ^10^citrate-synthase (CS, U/g prot); ^11^succinate dehydrogenase (SDH, U/g prot).

**Table 5:** Proximate composition of sea cucumber fed diets with different phosphorus levels.

	Dietary phosphorus level (%)						Pooled SE
	0.24	0.37	0.51	0.62	0.77	0.89	
Crude protein (%)	45.42 ± 0.65	44.70 ± 0.43	44.36 ± 0.38	44.61 ± 0.58	45.06 ± 0.19	45.51 ± 0.67	0.126
Crude fat (%)	4.04 ± 0.09	4.06 ± 0.12	4.05 ± 0.08	4.07 ± 0.09	4.13 ± 0.08	4.16 ± 0.08	0.021
Phospholipid (%)	0.46 ± 0.02^a^	0.52 ± 0.02^b^	0.56 ± 0.01^c^	0.58 ± 0.01^c^	0.58 ± 0.01^c^	0.59 ± 0.01^c^	0.011
Crude ash (%)	35.15 ± 0.45	34.90 ± 0.12	35.04 ± 0.13	35.24 ± 0.23	34.28 ± 0.37	35.35 ± 0.10	0.065
Phosphorus (mg/g)	1.78 ± 0.05^a^	1.88 ± 0.03^b^	1.96 ± 0.03^c^	2.00 ± 0.02^c^	2.01 ± 0.02^c^	2.02 ± 0.03^c^	0.022

Notes: values in the same row with different superscript letters show significant difference (*P* < 0.05).

**Table 6:** The intestinal oxidation resistance of sea cucumber fed diets with different phosphorus levels.

	Dietary phosphorus level (%)						Pooled SE
	0.24	0.37	0.51	0.62	0.77	0.89	
SOD^1^ (U/mg prot)	4.38 ± 0.30^a^	4.93 ± 0.09^bc^	5.26 ± 0.16^c^	5.24 ± 0.13^c^	4.96 ± 0.31^bc^	4.67 ± 0.17^ab^	0.085
CAT^2^ (U/mg prot)	6.11 ± 1.00	5.97 ± 0.72	6.00 ± 1.68	5.86 ± 0.35	5.89 ± 0.14	6.03 ± 0.20	0.108
GSH-Px^3^ (U/mg prot)	26.03 ± 1.26^a^	30.72 ± 2.29^bc^	33.36 ± 1.30^d^	33.52 ± 2.38^cd^	30.74 ± 1.54^bc^	27.51 ± 2.80^ab^	0.863
GSH^4^ (*μ*mol/g prot)	27.36 ± 2.05^a^	31.75 ± 1.65^b^	35.70 ± 2.20^bc^	34.79 ± 1.00^c^	34.47 ± 1.14^bc^	34.31 ± 1.49^bc^	0.691
GSSG^5^ (*μ*mol/g prot)	73.88 ± 1.32^a^	83.78 ± 10.53^b^	107.44 ± 13.34^c^	91.34 ± 12.79^bc^	79.87 ± 7.71^ab^	85.21 ± 17.55^bc^	4.232
GSH/GSSG	0.37 ± 0.03^a^	0.35 ± 0.01^a^	0.34 ± 0.03^a^	0.35 ± 0.01^a^	0.45 ± 0.01^b^	0.47 ± 0.04^b^	0.013
MDA^6^ (nmol/mg prot)	0.83 ± 0.02	0.80 ± 0.07	0.94 ± 0.09	0.95 ± 0.13	0.89 ± 0.10	0.84 ± 0.14	0.024

Notes: values in the same row with different superscript letters show significant difference (*P* < 0.05). ^1^Superoxide dismutase (SOD, U/mg prot); ^2^catalase (CAT, U/mg prot); ^3^glutathione peroxidase (GSH-Px, U/mg prot); ^4^glutathione (GSH, *μ*mol/g prot); ^5^glutathione oxidized (GSSG, *μ*mol/g prot); ^6^malondialdehyde (MDA, nmol/mg prot).

## Data Availability

The authors confirm that the data supporting the findings of this study are available within its supplementary material.

## Supplementary Materials

Supplementary Materials The data including growth, whole body composition, and the activities of intestinal enzymes of sea cucumber.
