# Supplementary material for: Influence of Dietary Phosphorus on the Growth, Feed Utilization, Proximate Composition, Intestinal Enzymes, and Oxidation Resistance of Sea Cucumber Apostichopus japonicus
Source: Aquac Nutr. 2023 Apr 20;2023:2266191. doi: 10.1155/2023/2266191 (PMC10139806; doi:10.1155/2023/2266191)
Supplement: Supplementary Materials — The data including growth, whole body composition, and the activities of intestinal enzymes of sea cucumber. [file 2266191.f1.docx]

**Growth and diet utizliation**

| P level (%) | IW (g) | FW (g) | WG (g) | SGR (%/d) | DFI (%/d) | DPI (10^-2^ %/d) | FE | FPR (%/d) |
| --- | --- | --- | --- | --- | --- | --- | --- | --- |
| 0.24 | 10 | 16.53333 | 6.533333 | 0.718276 | 2.100502513 | 0.504120603 | 0.334928 | 1.69921 |
| 0.24 | 9.966667 | 16.05333 | 6.086667 | 0.680958 | 2.074096849 | 0.497783244 | 0.322237 | 1.459317 |
| 0.24 | 10 | 16.81333 | 6.813333 | 0.742267 | 2.177697663 | 0.522647439 | 0.333383 | 1.788023 |
| 0.37 | 9.933333 | 19.29333 | 9.36 | 0.948376 | 2.477189781 | 0.916560219 | 0.369376 | 1.999153 |
| 0.37 | 10 | 19.18 | 9.18 | 0.930404 | 2.570253598 | 0.950993831 | 0.349714 | 2.079702 |
| 0.37 | 10.03333 | 19.44667 | 9.413333 | 0.945375 | 2.36996834 | 0.876888286 | 0.384951 | 1.954836 |
| 0.51 | 9.966667 | 20.9 | 10.93333 | 1.057861 | 2.60475162 | 1.328423326 | 0.388534 | 2.088244 |
| 0.51 | 10.06667 | 20.86 | 10.79333 | 1.040863 | 2.559862039 | 1.30552964 | 0.389527 | 2.140548 |
| 0.51 | 10 | 21.30667 | 11.30667 | 1.080621 | 2.494058773 | 1.271969974 | 0.413736 | 2.016001 |
| 0.62 | 10.1 | 21.34667 | 11.24667 | 1.069086 | 2.573669705 | 1.595675217 | 0.397035 | 2.158757 |
| 0.62 | 10 | 22.62667 | 12.62667 | 1.166492 | 2.568451165 | 1.592439722 | 0.430503 | 2.00975 |
| 0.62 | 9.733333 | 20.81333 | 11.08 | 1.085768 | 2.485814055 | 1.541204714 | 0.416907 | 1.938954 |
| 0.77 | 10 | 19.89333 | 9.893333 | 0.982571 | 2.223461195 | 1.71206512 | 0.425276 | 1.773926 |
| 0.77 | 10 | 20.05333 | 10.05333 | 0.994015 | 2.269299024 | 1.747360248 | 0.42117 | 1.838636 |
| 0.77 | 10 | 20.48 | 10.48 | 1.024091 | 2.292213473 | 1.765004374 | 0.428571 | 1.686352 |
| 0.89 | 10.03333 | 19.72 | 9.686667 | 0.965315 | 2.177907237 | 1.938337441 | 0.427102 | 1.518197 |
| 0.89 | 10.03333 | 19.34667 | 9.313333 | 0.93801 | 2.108009984 | 1.876128886 | 0.429648 | 1.661966 |
| 0.89 | 9.966667 | 19.60667 | 9.64 | 0.966605 | 2.181939502 | 1.941926157 | 0.421145 | 1.534698 |

**Digestibility**

| P level (%) | dry material (%) | Protein (%) | Energy (%) | Fat (%) | Phorpherous (%) |
| --- | --- | --- | --- | --- | --- |
| 0.24 | 17.18 | 21.67 | 28.75 | 33.25 | 34.88 |
| 0.24 | 18.72 | 24.28 | 30.37 | 31.15 | 33.69 |
| 0.24 | 20.33 | 22.33 | 29.28 | 38.34 | 32.52 |
| 0.37 | 18.87 | 22.36 | 29.58 | 34.28 | 32.25 |
| 0.37 | 19.09 | 23.15 | 27.66 | 33.19 | 32.08 |
| 0.37 | 16.83 | 20.67 | 28.29 | 31.28 | 33.22 |
| 0.51 | 17.21 | 23.18 | 26.08 | 33.21 | 30.79 |
| 0.51 | 19.23 | 24.19 | 25.05 | 30.37 | 31.98 |
| 0.51 | 16.24 | 22.67 | 26.42 | 31.76 | 30.69 |
| 0.62 | 18.89 | 25.55 | 27.06 | 32.14 | 29.26 |
| 0.62 | 20.17 | 24.18 | 28.78 | 34.28 | 30.17 |
| 0.62 | 18.42 | 21.62 | 28.12 | 34.09 | 33.33 |
| 0.77 | 21.66 | 24.37 | 33.25 | 33.23 | 27.17 |
| 0.77 | 18.89 | 22.91 | 32.18 | 36.85 | 25.64 |
| 0.77 | 19.38 | 20.25 | 27.27 | 32.19 | 26.71 |
| 0.89 | 21.01 | 23.25 | 34.56 | 34.28 | 23.51 |
| 0.89 | 19.95 | 20.86 | 30.1 | 36.09 | 26.36 |
| 0.89 | 21.22 | 24.37 | 32.38 | 33.52 | 25.27 |

**Whole-body composition**

| P level (%) | Protein (%) | Fat (%) | Phospholipid (%) | Ash (%) | Phorpherous (%) |
| --- | --- | --- | --- | --- | --- |
| 0.24 | 45.32 | 4.04 | 0.44 | 35.66 | 1.728 |
| 0.24 | 44.78 | 4.12 | 0.46 | 34.82 | 1.775 |
| 0.24 | 45.07 | 3.95 | 0.48 | 34.98 | 1.827 |
| 0.37 | 44.87 | 4.19 | 0.51 | 34.79 | 1.906 |
| 0.37 | 45.01 | 3.96 | 0.55 | 34.88 | 1.886 |
| 0.37 | 44.21 | 4.03 | 0.51 | 35.03 | 1.851 |
| 0.51 | 45.16 | 4.05 | 0.57 | 34.91 | 1.938 |
| 0.51 | 45.12 | 3.97 | 0.58 | 35.04 | 1.945 |
| 0.51 | 45.79 | 4.12 | 0.55 | 35.17 | 1.989 |
| 0.62 | 46.18 | 4.18 | 0.58 | 35.51 | 1.998 |
| 0.62 | 45.02 | 4.01 | 0.59 | 35.09 | 1.977 |
| 0.62 | 45.63 | 4.03 | 0.58 | 35.13 | 2.021 |
| 0.77 | 45.17 | 4.1 | 0.59 | 34.97 | 1.986 |
| 0.77 | 44.84 | 4.22 | 0.59 | 35.69 | 2.034 |
| 0.77 | 45.16 | 4.07 | 0.589 | 35.18 | 2.007 |
| 0.89 | 46.27 | 4.09 | 0.59 | 35.37 | 2.05 |
| 0.89 | 44.99 | 4.25 | 0.59 | 35.24 | 1.988 |
| 0.89 | 45.27 | 4.13 | 0.58 | 35.43 | 2.013 |

**Intestinal enzyme activity**

| P level (%) | ACPT (U/mg prot) | AKPT (U/mg prot) | Amylase (U/mg prot) | lipase (U/g prot) | ALP(U/g prot) | HK(U/g prot) | PFK(U/g prot) | AST(U/g prot) | ALT(U/g prot) | PK(U/g prot) | PEPCK(U/g prot) | CS(U/g prot) | SDH(U/g prot) |
| --- | --- | --- | --- | --- | --- | --- | --- | --- | --- | --- | --- | --- | --- |
| 0.24 | 0.538674 | 223.0366 | 16.29455 | 4.027451 | 53.03404 | 69.657 | 8.952148 | 978.397 | 3939.502 | 26.93402 | 41.22184 | 5.048307 | 5.087259 |
| 0.24 | 0.5464 | 241.3624 | 16.50989 | 5.266667 | 58.13243 | 63.7803 | 9.58536 | 1087.464 | 3884.179 | 31.34734 | 49.17039 | 5.823222 | 5.694691 |
| 0.24 | 0.592829 | 266.4254 | 15.11138 | 5.603922 | 53.42714 | 69.73316 | 7.728269 | 818.8471 | 4051.494 | 27.57066 | 46.09062 | 5.077733 | 5.138919 |
| 0.37 | 0.570065 | 224.6879 | 18.53804 | 7.12549 | 73.33549 | 77.91384 | 9.362445 | 653.1739 | 3355.884 | 32.9758 | 44.5567 | 5.672793 | 5.942286 |
| 0.37 | 0.486311 | 213.4958 | 17.25328 | 8.054902 | 70.04992 | 71.99161 | 9.98484 | 748.5234 | 3111.166 | 31.55719 | 45.4469 | 6.72884 | 5.545152 |
| 0.37 | 0.596941 | 223.7064 | 17.97574 | 7.647059 | 72.84129 | 82.30302 | 8.289085 | 650.2496 | 3128.699 | 32.01968 | 42.49084 | 4.127716 | 6.217583 |
| 0.51 | 0.655168 | 193.6341 | 18.13043 | 6.815686 | 83.69944 | 68.82212 | 13.05901 | 621.8045 | 3066.289 | 40.50629 | 28.07366 | 4.245891 | 5.944602 |
| 0.51 | 0.637022 | 201.9879 | 20.57983 | 7.12549 | 82.28514 | 73.86283 | 10.73846 | 581.4421 | 3083.192 | 44.1299 | 30.15672 | 4.863837 | 6.526295 |
| 0.51 | 0.482213 | 202.131 | 20.08155 | 6.913725 | 84.25666 | 64.41814 | 12.23425 | 602.7529 | 3420.668 | 42.74972 | 27.26119 | 5.539816 | 6.575204 |
| 0.62 | 0.502248 | 144.3275 | 18.44175 | 7.266667 | 82.21998 | 64.13438 | 14.86441 | 590.3003 | 2984.829 | 43.57378 | 25.95992 | 5.418672 | 6.879214 |
| 0.62 | 0.470866 | 161.1606 | 23.60517 | 7.956863 | 83.4126 | 58.12153 | 12.27051 | 485.6965 | 2675.451 | 41.09594 | 24.24735 | 4.375829 | 6.26767 |
| 0.62 | 0.511073 | 163.5851 | 21.90835 | 6.815686 | 87.26754 | 58.09836 | 11.75426 | 611.7208 | 3186.406 | 46.61683 | 26.84295 | 4.726916 | 5.610684 |
| 0.77 | 0.498292 | 163.2514 | 22.91511 | 6.505882 | 68.49618 | 65.87766 | 12.21395 | 515.1908 | 2675.594 | 39.57918 | 30.9936 | 5.56412 | 5.114366 |
| 0.77 | 0.500018 | 138.7405 | 20.13143 | 8.364706 | 78.28627 | 64.62063 | 10.54756 | 504.2662 | 3001.517 | 37.8698 | 27.25795 | 5.114292 | 5.459571 |
| 0.77 | 0.52517 | 150.6689 | 19.48742 | 7.435294 | 72.02412 | 62.14579 | 9.943741 | 590.4099 | 2812.938 | 40.71032 | 26.90591 | 4.751677 | 5.115419 |
| 0.89 | 0.54316 | 145.1755 | 19.1313 | 5.886275 | 64.87867 | 65.8526 | 7.118864 | 524.3497 | 2911.352 | 30.47856 | 32.07361 | 4.778966 | 5.082326 |
| 0.89 | 0.53114 | 124.7063 | 18.2873 | 6.402614 | 67.59438 | 64.44231 | 7.795211 | 519.4474 | 2326.195 | 32.70346 | 33.17288 | 4.742395 | 4.569633 |
| 0.89 | 0.588252 | 146.6344 | 21.72346 | 8.647059 | 67.57258 | 58.44869 | 6.807767 | 454.393 | 3005.28 | 38.3159 | 33.10825 | 5.340825 | 4.821816 |

**Intestinal oxidation resistance**

| P level  (%) | SOD  (U/mg prot) | GSH  (μmol/g prot) | GSSG  (μmol/g prot) | GSGH/GSSG | GSH-px (U/mg prot) | CAT (U/mg prot) | MDA  (nmol/mg prot) |
| --- | --- | --- | --- | --- | --- | --- | --- |
| 0.24 | 4.72 | 27.2701 | 72.7174 | 0.375 | 24.65 | 5.65 | 0.8047 |
| 0.24 | 4.26 | 25.3606 | 75.3119 | 0.3367 | 26.32 | 7.25 | 0.8536 |
| 0.24 | 4.17 | 29.4535 | 73.6354 | 0.4 | 27.12 | 5.42 | 0.8275 |
| 0.37 | 4.72 | 31.0762 | 89.5099 | 0.3472 | 30.12 | 5.84 | 0.7609 |
| 0.37 | 4.49 | 33.6399 | 91.6299 | 0.3671 | 28.79 | 6.75 | 0.7586 |
| 0.37 | 4.81 | 30.5451 | 90.1978 | 0.3386 | 33.25 | 5.32 | 0.8857 |
| 0.51 | 5.18 | 33.5788 | 97.5462 | 0.3442 | 37.15 | 6.11 | 0.8542 |
| 0.51 | 5.44 | 31.9652 | 102.1678 | 0.3129 | 33.31 | 6.09 | 1.0391 |
| 0.51 | 5.15 | 34.5466 | 92.6063 | 0.373 | 34.52 | 5.81 | 0.9194 |
| 0.62 | 5.09 | 34.6758 | 104.181 | 0.3328 | 36.17 | 5.49 | 0.9266 |
| 0.62 | 5.35 | 33.8557 | 94.8132 | 0.3571 | 31.56 | 6.18 | 0.8395 |
| 0.62 | 5.28 | 35.8382 | 102.0422 | 0.3512 | 32.83 | 5.90 | 1.0864 |
| 0.77 | 4.6 | 34.8235 | 78.4425 | 0.4439 | 32.48 | 5.77 | 0.9686 |
| 0.77 | 5.14 | 33.1971 | 73.4927 | 0.4517 | 30.17 | 5.86 | 0.9332 |
| 0.77 | 5.15 | 35.3909 | 77.6806 | 0.4556 | 29.56 | 6.05 | 0.7816 |
| 0.89 | 5.02 | 34.486 | 78.475 | 0.4395 | 27.85 | 6.22 | 0.84 |
| 0.89 | 4.85 | 35.692 | 68.9557 | 0.5176 | 30.13 | 6.03 | 0.7068 |
| 0.89 | 4.93 | 32.7378 | 73.8191 | 0.4435 | 24.56 | 5.83 | 0.9864 |
